# Machine Learning Prediction of Estimated Risk for Bipolar Disorders Using Hippocampal Subfield and Amygdala Nuclei Volumes

**DOI:** 10.3390/brainsci13060870

**Published:** 2023-05-27

**Authors:** Fabian Huth, Leonardo Tozzi, Michael Marxen, Philipp Riedel, Kyra Bröckel, Julia Martini, Christina Berndt, Cathrin Sauer, Christoph Vogelbacher, Andreas Jansen, Tilo Kircher, Irina Falkenberg, Florian Thomas-Odenthal, Martin Lambert, Vivien Kraft, Gregor Leicht, Christoph Mulert, Andreas J. Fallgatter, Thomas Ethofer, Anne Rau, Karolina Leopold, Andreas Bechdolf, Andreas Reif, Silke Matura, Silvia Biere, Felix Bermpohl, Jana Fiebig, Thomas Stamm, Christoph U. Correll, Georg Juckel, Vera Flasbeck, Philipp Ritter, Michael Bauer, Andrea Pfennig, Pavol Mikolas

**Affiliations:** 1 Department of Psychiatry and Psychotherapy, Carl Gustav Carus University Hospital, Technische Universität Dresden, 01062 Dresden, Germany; 2 Department of Psychiatry and Behavioral Sciences, Stanford University School of Medicine, Stanford, CA 94305, USA; 3 Core-Facility Brainimaging, Faculty of Medicine, University of Marburg, 35037 Marburg, Germany; 4 Translational Clinical Psychology, Philipps-University Marburg, 35037 Marburg, Germany; 5 Center for Mind, Brain and Behavior (CMBB), University of Marburg and University Giessen, 35039 Marburg, Germany; 6 Department of Psychiatry and Psychotherapy, University of Marburg, 35037 Marburg, Germany; 7 Department of Psychiatry and Psychotherapy, University Medical Center Hamburg-Eppendorf, 20251 Hamburg, Germany; 8 Centre for Psychiatry, Justus-Liebig University Giessen, 35390 Gießen, Germany; 9 Department of Psychiatry, Tuebingen Center for Mental Health, University of Tuebingen, 72074 Tuebingen, Germany; 10 Department of Psychiatry, Psychotherapy and Psychosomatic Medicine, Vivantes Hospital Am Urban and Vivantes Hospital Im Friedrichshain, Charité-Universitätsmedizin, 10117 Berlin, Germany; 11 Department of Psychiatry, Psychosomatic Medicine and Psychotherapy, Goethe University Frankfurt, University Hospital, 60323 Frankfurt, Germany; 12 Department of Psychiatry and Psychotherapy, Charité Campus Mitte, Charité University Medicine, 10117 Berlin, Germany; 13 Department of Clinical Psychiatry and Psychotherapy, Brandenburg Medical School Theodor Fontane, 16816 Neuruppin, Germany; 14 Department of Child and Adolescent Psychiatry, Charité Universitätsmedizin Berlin, 10117 Berlin, Germany; 15 Department of Psychiatry, Northwell Health, The Zucker Hillside Hospital, Glen Oaks, New York, NY 11004, USA; 16 Department of Psychiatry and Molecular Medicine, Donald and Barbara Zucker School of Medicine at Hofstra/Northwell, Hempstead, NY 11549, USA; 17 Department of Psychiatry, Psychotherapy and Preventive Medicine, LWL University Hospital, Ruhr-University, 44791 Bochum, Germany

**Keywords:** bipolar risk, hippocampal subfields, amygdala nuclei, MRI, machine learning

## Abstract

The pathophysiology of bipolar disorder (BD) remains mostly unclear. Yet, a valid biomarker is necessary to improve upon the early detection of this serious disorder. Patients with manifest BD display reduced volumes of the hippocampal subfields and amygdala nuclei. In this pre-registered analysis, we used structural MRI (*n* = 271, 7 sites) to compare volumes of hippocampus, amygdala and their subfields/nuclei between help-seeking subjects divided into risk groups for BD as estimated by BPSS-P, BARS and EPI*bipolar*. We performed between-group comparisons using linear mixed effects models for all three risk assessment tools. Additionally, we aimed to differentiate the risk groups using a linear support vector machine. We found no significant volume differences between the risk groups for all limbic structures during the main analysis. However, the SVM could still classify subjects at risk according to BPSS-P criteria with a balanced accuracy of 66.90% (95% *CI* 59.2–74.6) for 10-fold cross-validation and 61.9% (95% *CI* 52.0–71.9) for leave-one-site-out. Structural alterations of the hippocampus and amygdala may not be as pronounced in young people at risk; nonetheless, machine learning can predict the estimated risk for BD above chance. This suggests that neural changes may not merely be a consequence of BD and may have prognostic clinical value.

## 1. Introduction

Bipolar disorders (BD) are serious recurrent, chronic mental disorders starting in early adulthood (18.4–20 years) [1], which contribute to 6.8% (4.9–9.1) disability-adjusted life years lost due to mental disorders [2]. A longer duration of untreated illness leads to more depressive and manic episodes and more suicidal behavior [3]. Ten to twenty percent of patients suffering from BD commit suicide throughout their disease course [4]. Hence, early detection and treatment is crucial.

Detection of BD risk prior to diagnosis has been increasingly subject to scientific research [5]. One established approach is to study genetic risk at the individual level, i.e., BD diagnoses among first-degree relatives [6]. The transition rates of first-degree relatives have been estimated at 4.2–22.4% [7,8,9]. Another approach is to study a broader range of clinical risk factors in help-seeking populations using risk assessment tools. For instance, the prognostic accuracies of two clinical interviews have been investigated: The Bipolar At-Risk States Revised (BARS; Harrell’s *C* = 0.777) and the Semistructured Interview of Bipolar At-Risk States (SIBARS; Harrell’s *C* = 0.742) [10]. Besides SIBARS, there are two other established BD risk assessment tools that we used here: the Early Phase Inventory for Bipolar Disorders (EPI*bipolar* [11]), a semi-structured interview consisting of a broad range of literature-derived risk factors for BD and the Bipolar Prodrome Symptom Scale—Prospective (BPSS-P [12]), a semi-structured interview assessing three different scales based on DSM IV criteria. Besides interviews, using biomarkers, such as structural neuroimaging, might also yield a good prognostic accuracy and thus lead to earlier detection of psychiatric disorders and better clinical outcomes [13,14].

During the search for such biomarkers, several structural abnormalities have already been identified in BD [15]. These include a reduced cortical thickness in frontal, parietal and temporal regions [16], as well as smaller subcortical structures (hippocampus, thalamus and amygdala) [17]. By using more fine-grained parcellation methods, it became possible to study subcortical structures and their subregions. For instance, the hippocampus is a region of interest (ROI) that has been investigated thoroughly in an important mega-analysis with 4698 subjects: the authors found significantly smaller volumes of the hippocampus and some of its subfields (whole hippocampus, GC-ML-DG, CA4, CA3, CA1, subiculum, presubiculum, molecular layer HP, HATA and hippocampal tail) but not others (parasubiculum, fimbria and hippocampal fissure) in BD patients compared to healthy controls (HC) [18]. Another study found some subfields to be smaller in both BD and schizophrenia (SZ) patients, compared to HC (bilateral CA2/3, CA4/dentate gyrus, subiculum and right CA1), whereas presubiculum volumes were only smaller in SZ [19]. In an investigation of different psychotic disorders, smaller subfield volumes in psychotic BD could be found only in the bilateral CA2/3, the left presubiculum and the right CA4/DG, compared to HC [20].

Another valuable ROI for early detection of BD, with less BD-specific scientific literature available to date, is the amygdala and its nuclei. The amygdala is involved in emotion regulation via connections with the medial prefrontal and the orbitofrontal cortex, the inferior frontal gyrus, the hypothalamus and the ventromedial striatum [21,22]. Its nuclei show widespread but differentially organized connections. A large-scale multicentric study in 1710 BD patients and 2594 HC revealed smaller total amygdala volumes only for BD I patients [17]. More specifically, Barth et al. [23] found a decreased volume of the basal nucleus, accessory basal nucleus, anterior amygdaloid area and cortico-amygdaloid transition area in BD I, but only a smaller volume of the basal nucleus and the cortico-amygdaloid transition area in BD II, both compared to HC. On the other hand, Bielau et al. [24] found no significant differences in the total amygdala volume in a post-mortem study between BD patients and HC. Thus, reduced volumes of the segmented nuclei of the amygdala, rather than total volume, might be considered a potential risk factor for BD. Besides these structural alterations often found, there are several studies pointing out functional alterations for both the amygdala [25,26] and the hippocampus [27,28], which further highlights their important role in the pathophysiology of BD. Although these regions have been thoroughly analyzed in manifest BD, they have not been the object of investigation in individuals at risk. It is not understood if the reduction in the volume of the above-mentioned structures represents a risk factor for the development of the disease or, rather, its long-term consequence. The hippocampus is one of the most plastic brain areas: for example, in one study, its volume was larger in long-term meditators compared to controls in many subfields, even up to 15% [29]. Interestingly, BD patients treated with mood stabilizers (i.e., taking lithium over 24 months) have larger amygdala and hippocampus volumes than untreated BD patients and cannot be differentiated from HC [30]. Indeed, both structures may react sensitively to environmental factors not only via neuroproliferation but also via neurogenesis with comparable cell turnover rates [31]. Neurogenesis, particularly in the hippocampus, is, for instance, strongly affected by chronic stress via different mechanisms, such as neurotoxic effects of glucocorticoids, glial change or shrinkage of apical dendrites [32]. Simultaneously, the stress response is altered in BD: Patients who experience chronic stress might be more vulnerable to acute stress due to dysregulations in their endocrine and immune responses [33]. Thus, environmental factors, neuroplasticity and pathophysiology of BD are interlinked. An analysis of hippocampal subfields and amygdala nuclei in individuals at risk for BD might help us to understand the role of these brain alterations throughout the course of the disease.

While group comparisons using null hypothesis testing can show mean structural abnormalities, they often cannot be used to draw inferences about individuals [34]. On the other hand, multivariate machine learning (ML) approaches have a higher sensitivity and can potentially improve individual inferences required for clinical diagnostics and prognostics. We have already investigated the use of ML in combination with regional cortical thickness and surface area values as well as subcortical structural volumes (Mikolas et al., 2023) [35]. Here, we classified subjects at risk of developing BD according to the BPSS-P criteria and achieved a balanced accuracy of 63.1 % using a 10-fold cross-validation.

In this pre-registered analysis of the same sample of help-seeking individuals with risk factors for BD, we first analyzed volume differences of hippocampal subfields and amygdala nuclei using statistical between-group comparisons. As the main analysis, we used a linear support vector machine (SVM) to classify subjects into different BD risk groups. SVMs are a widely used algorithm in medical research [36]. The risk stratification into risk states/syndromes for BD was performed using three assessment tools: BARS [10], EPI*bipolar* [11] and BPSS-P [12].

## 2. Materials and Methods

### 2.1. Pre-Registration

The study was pre-registered via the open science framework as a registration following analysis of the data. The pre-registration was updated on 23 October 2022 due to minor errors in the protocol. For detailed information, please see https://osf.io/xz9vt (accessed on 26 May 2023).

### 2.2. Sample

The data was collected as part of the Early-BipoLife project [37,38]. Early-BipoLife is a multicentric, naturalistic, prospective-longitudinal observational cohort study of adolescents and young adults (age 15–35 years) at risk for BD. It was performed at ten German university centers and teaching hospitals with early detection facilities. The subjects were recruited by psychological staff in early detection centers (six study centers), psychiatric in- and outpatient clinics (nine study centers) and through local advertising (two study centers). For this analysis, we only used the data acquired at baseline. MRI acquisitions were performed on seven of the ten study sites: Berlin, Bochum, Frankfurt, Hamburg, Dresden, Marburg and Tübingen. Of the total *n* = 1229 recruited adolescents and young adults included in this study, *n* = 313 opted to receive an MRI. For a detailed look at final sample sizes, see 2.6 Quality control and data exclusion.

The recruitment process comprised three different pathways with the following inclusion criteria: In the first pathway (*n* = 123), subjects consulted an early recognition center and had a presence of at least one BD risk factor (family BD history, (sub)threshold affective symptomatology/depressive syndrome, hypomanic/mood swings, disturbances of circadian rhythm/sleep and other clinical hints). The participants who took part in the study via early detection centers approached the centers either out of their own interest due to psychological distress or after being referred by a psychiatrist or psychotherapist for specific diagnostics regarding bipolar disorder. The second pathway (*n* = 146) comprised in- or outpatients with a depressive syndrome (major depressive disorder, dysthymic disorder, cyclothymic disorder, minor depressive disorder, recurrent brief depressive disorder, adjustment disorder with depressed mood, depressive disorder not otherwise specified). The third pathway (*n* = 44) comprised in- or outpatients with a clinically confirmed ADHD diagnosis. ADHD patients were included since the combined subtype of ADHD, with both inattentive and hyperactive/impulsive symptoms, was previously associated with BD [11]. For complete inclusion and exclusion criteria, see Appendix A. As the reasons for consultation might have generally differed between the ‘early recognition’ pathway and the two other ones, we included the recruitment pathway as a confounder in all analyses.

The age criterion was based on available studies on age of onset and time to diagnosis. About 75% of individuals with BD I would develop the disorder before the age of 42 years [39], and about 70% would develop any BD by the age of 21 [1]. On the other hand, in the health care system of Germany, it takes 12.4 years on average to establish the diagnosis since the onset of first symptoms [40]. Failure to establish the correct diagnoses occurs mainly due to the predominance of depressive symptoms, as well as unrecognized hypomania [1,12,41]. Thus, we extended the age criterion to 35 years to include older individuals with unrecognized BD.

Informed consent was obtained from all subjects involved in the study. Additionally, parents of adolescents gave their informed consent about their children’s participation. The study was approved by the Ethics Committee of the Medical Faculty of the Technische Universität Dresden (No: EK290082014, 05.02.2015), as well as local ethics committees at each study site.

### 2.3. Risk Assessment Instruments

We assessed the risk state for BD with three independent assessment tools. The Semistructured Interview for Bipolar At-Risk States (SIBARS [10]) is an assessment tool developed for young people aged 15–35 covering five different subscales: subthreshold mania, depression, cyclothymic features, genetic risk and mood swings. While cyclothymic features and genetic risk are binary categories, the three other scales are measured with both a severity and a frequency score. The interview items for these subscales were adapted from established rating scales, and decisions about inclusion were made with the clinical expertise of the authors [10]. Experienced clinicians had training on how to apply the instrument and discussed the scoring under supervision. The instrument achieved a prognostic accuracy of Harrell’s *C* = 0.742 in a sample of subjects with a high risk for psychosis [10].

The Early Phase Inventory for Bipolar Disorders (EPI*bipolar* [11]) is a semi-structured interview that was developed based on a systematic literature review. It covers a broad range of risk factors for BD. To quantify the BD risk, all risk factors are weighted and classified into either primary symptoms (such as genetic risk, increasing cyclothymia dynamic and prodromal (hypo-)manic symptoms) or secondary symptoms (such as specific changes in sleep and circadian rhythm, substance use, (suspected) diagnosis of ADHD, impairment of psychosocial functioning, manifest or earlier affective disorder other than BD, or fearfulness/anxiety). Finally, subjects are categorized into four different risk groups: no risk at present, risk status (at least one secondary symptom accompanied by specific changes in sleep and circadian rhythm), high-risk status (one primary and at least one secondary symptom) and ultra-high risk (more than one primary symptom). In later analyses, the high-risk and ultra-high-risk groups were fused since the high-risk group contained a disproportionally low number of subjects (3.2%) [42]. Therefore, we used the EPI*bipolar* version with three risk categories: no risk, low-risk and high-risk across this manuscript.

The Bipolar Prodrome Symptom Scale—Prospective BPSS-P [12] is a semi-structured interview assessing three scales: mania (ten questions), depression (twelve questions) and a general symptom index (nine questions). Each question is rated between absent (zero) and extreme (six). The symptoms and scales are based on DSM-IV criteria and other established rating scales. Internal consistency was Cronbach’s α = 0.87 for mania, α = 0.89 for depression and α = 0.74 for the general symptom index [12]. Additionally, it showed good inter-rater reliability (*ICC* = 0.939 for the total score) as well as convergent validity to comparable scales [12].

### 2.4. MRI Acquisition and Preprocessing

All seven utilized MRI scanners had a field strength of 3 Tesla. Six of the seven were manufactured by Siemens, and one scanner in Bochum by Philips. For a detailed explanation of hardware, software and scanner protocols, please see the study protocol by Vogelbacher et al. [43].

T1-weighted structural scans were preprocessed using Freesurfer 7.1.1. [44,45,46,47] running on the Centre for Information Services and High-Performance Computing (ZIH) by TU Dresden (https://tu-dresden.de/zih/hochleistungsrechnen (accessed on 26 May 2023). The ZIH offers a virtual environment specifically designed for parallel, data-intensive applications, holding about 60,000 cores. Here, we wrote a bash shell prompting parallel processing within Freesurfer 7.1.1 for all our subjects in a virtual Linux environment (see Appendix B). Since the ZIH did not hold the required MATLAB runtime necessary for the Freesurfer pipeline segmentation of hippocampal subfields and nuclei of the amygdala, we had to run this part on a remote computer. Here, we only could install Freesurfer 7.2.0 using Windows Subsystem for Linux (WSL 2). However, the automated segmentation pipeline is the same for all versions of Freesurfer 7 or higher. Thus, this change in software version did not have any effect on our processed data. The segmentation process is a widely used algorithm for computing probabilistic atlases to localize the independent nuclei and subfields for each subject [46,47,48]. These statistical atlases were obtained from ultra-high resolution, ex vivo MRI scans. After a first manual segmentation and a manual annotation of adjacent areas using T1-weighted MRI scans, the developers of the software built a computational atlas using Bayesian inference [46]. Each node of this computational atlas contains probabilistic information about its assignment to each relevant subregion [49]. The segmentation of a previous unfamiliar dataset is then solved as “[…] a Bayesian inference problem of maximizing the probability of the segmentation—given the atlas and the input image” [50]. Summarized, we decided to use the Freesurfer segmentation pipeline to (A) enhance reproducibility to other BD study groups, for instance, ENIGMA, and (B) due to its good reliability metrics, such as an overall high test–retest reliability, with an *ICC* larger than 0.5 and most studies reporting an *ICC* around 0.9 [50]. For a detailed explanation of quality control, see below.

### 2.5. Measured Variables

We assessed the risk for BD using three independent tools. Results were binary variables, no risk vs. risk for BARS and BPSS-P and three risk groups for EPI*bipolar*. Further, we estimated the volumes of hippocampal subfields and nuclei of the amygdala with the above-explained pipeline. The output consists of three main outputs, twelve hippocampal subfields and nine nuclei of the amygdala. For an overview, see Table 1. For an overview of the graphical representation of the parcellations, we recommend Tesli et al. (2020) [51]. For the medical history, we collected data on psychiatric medication at baseline (yes/no for five classes of medication: antidepressants, antipsychotics, mood stabilizers, anxiolytics and hypnotics and psychostimulants). For the analysis, we counted the binary variable medication as yes if a subject was positive on at least one medication class. As only seven subjects took lithium (each under one year), we could not further analyze its potential effect on the subcortical volumes during risk state. Additionally, we collected the smoking status (never smoked/current smoker/past smoker). Data on present and lifetime use of cannabis was collected since it previously was associated with a smaller hippocampus [52] (no use/<1× month/~1× month/2–9× month/≥10× month). Early life adversities are typically associated with alterations of hippocampal subfield volumes [53,54,55,56]. We estimated it using the total score of the Child Trauma Questionnaire (CTQ) questionnaire, which consists of 25 five-point Likert scale items with five subscales on different traumatic experiences and three extra items on trivialization [57].

### 2.6. Quality Control and Data Exclusion

For quality assurance, the MRI images were analyzed using the MRIQC tool [58]. For this dataset, a visual inspection by two authors was performed. A set of *n* = 23 subjects was excluded from further analysis due to strong movement, ghosting or fold-over artifacts, which gives us remaining *n* = 290. The specific MRI quality control protocol is described elsewhere [43]. Since the data was already collected during the Early-BipoLife project, we generated our hypotheses after first acquisition of data, which is a legitimate approach and which we explicitly stated in the registration (see above). We kept our workflow independent from any preliminary results to achieve good scientific practice. This was possible since the MRI data we used here (segmented hippocampal subfields and nuclei of the amygdala) had not been preprocessed before and thus was not accessible to us in advance of hypothesis generation.

After preprocessing the T1 images, we performed a standardized quality control of the segmentation of hippocampal subfields and nuclei of the amygdala according to the established protocols of the ENIGMA working group (http://enigma.ini.usc.edu/protocols/imaging-protocols (accessed on 26 May 2023). Thus, we ran an RStudio script (RStudio Team, 2022.07.2, Boston, MA, USA) computing outliers and a ranking analysis for the subfield and nuclei volumes. After generating an HTML file containing several pictures for each subject and hemisphere in three different planes, two investigators performed visual quality control independently. The focus was on the subjects statistically identified as outliers. Here, we discarded *n* = 4 subjects: one due to failed preprocessing and three because of major segmentation errors. Other reasons for exclusion were failed preprocessing (*n* = 4), drop-out (*n* = 4), no longer wishing to participate (*n* = 2), baseline data incomplete (*n* = 1) and missing data in the three respective risk assessment tools (*n* = 11 for BPSS-P, *n* = 13 for BARS and *n*= 4 for EPI*bipolar*). This resulted in final sample sizes of *n* = 264 (BPSS-P), *n* = 262 (BARS) and *n* = 271 (EPI*bipolar*). For the covariates, we imputed the sample mean (continuous variables) or mode (discrete variables) using IBM SPSS Statistics (Version 28.0.1.1, Armonk, NY, USA) since both LME and SVM cannot handle missing data. This was the case for smoking status (*n* = 2), lifetime cannabis use (*n* = 3), CTQ score (*n* = 13) and psychiatric medication (*n* = 4). The covariates gender, age, present cannabis use, education and study site were available for all subjects.

### 2.7. Statistical Analysis

We compared demographic and clinical variables between the respective risk score groups (low vs. high risk for BPSS-P and BARS, no risk vs. low-risk vs. high-risk for EPI*bipolar*) using *χ2* analysis for categorical data and t-tests or Wilcoxon rank-sum tests for continuous data (depending on whether the data are normally distributed or not). Then, we tested if the volumes of the whole hippocampus and the whole amygdala differed significantly between high and low BD risk scores. To achieve this, we calculated six independent models: one for each combination of the three risk assessment tools with hippocampus and amygdala, respectively. To achieve reproducibility, we followed the statistical analysis pipeline from a previously published paper on hippocampal subfields in BD patients versus HC, where they also used a linear mixed effects model [16]. For the three LME models predicting the hippocampus, we included its total volume as a dependent variable and BD risk score (binary factor for BPSS-P and BARS and three-category factor for EPI*bipolar*) as a fixed effect variable. Sex, age, sex×age (factors, fixed), scanner site (factor, random), CTQ total (continuous), present and lifetime cannabis use (continuous), ICV (continuous) and medication (binary) were included as covariates. Sex, age and their interaction were included because the volume of the hippocampus follows a non-linear age-related trend [59], its subfields differ between men and women even after adjusting for total hippocampal volume and brain size [60], and the sex differences in age-related volume changes are not so clear yet. The same procedure was followed for the amygdala, where its total volume was used as a dependent variable. We further followed the previously published study and averaged the volumes of both hemispheres since there was no theoretical consideration of potential lateral differences and to reduce number of tests [18].

For each significant model, we performed post hoc LME models with above-mentioned covariates and variables of interest using each of the twelve subfields (in case of hippocampus) or each of the nine nuclei (in the case of amygdala). To achieve a balance between reducing false-positive results and gaining an acceptable test power, we corrected for multiple testing using the false discovery rate (FDR by Benjamini and Hochberg [61]). We considered FDR *q* < 0.05 as significant. All LME models were calculated using IBM SPSS Statistics (Version 28.0.1.1, Armonk, NY, USA), while the FDR correction was performed with a MATLAB script (R2022a, Natick, MA, USA), see Appendix C. Since the three risk assessment tools differ, we decided to correct for each tool independently. Thus, we corrected for two *p*-values during the main analysis (total hippocampus and amygdala volumes) and 21 *p*-values during the explorative analysis (twelve hippocampal subfields and nine nuclei of the amygdala volumes) for each tool, respectively.

### 2.8. Machine Learning Analysis

To increase reproducibility and comparability with a previous study of structural MRI in BD by the ENIGMA consortium, we used a linear support vector machine (SVM) [62]. An advantage of a linear SVM is that its coefficients can be interpreted as relative measures of feature importance, which allows for explainable models [62]. A linear SVM has one single hyperparameter C, which determines whether the algorithm allows more misclassifications to avoid overfitting the training data (lower C) or fewer misclassifications to enhance the accuracy (higher C) [63]. We optimized C using nested cross-validation and a grid search method using the following values for C: 10^-5^, 10^-4^, 10^-3^, 10^-2^, 10^-1^, 1, 10 and 100. As the subjects completed three risk assessment tools, we trained three independent SVMs. We used the volumes of 18 amygdala nuclei (right and left), 24 volumes of hippocampal subfields (right and left) and total volumes of amygdala and hippocampus (right and left), i.e., total of 44 features. To evaluate the SVM performance, we used 10-fold cross-validation, i.e., we divided the sample into ten independent subsets of similar size and trained the model ten times, using all the data of the subsamples except for one, which was left out as validation set [64]. To evaluate the generalizability across study sites, we additionally performed leave-one-site-out cross-validation, training the classifier on the data of six sites and testing the data from one left-out site in each fold. To account for the imbalanced class distribution within the data, we (A) kept the class ratio in all folds approximately the same (i.e., stratified cross-validation), and (B) we used random oversampling of the minority class [65] in the training set so that the class ratios in each fold was balanced. To preserve the train/test separation, we standardized the features separately in the training and testing sets by removing the mean and scaling to unit variance. We evaluated the performance using standard metrics for imbalanced datasets (balanced accuracy, Cohen’s kappa, sensitivity, specificity). We report the performance results as the average value on all folds. Similarly, we computed 95% confidence intervals based on the performance results on all folds. We performed binary classifications for each risk assessment instrument separately between subjects who fulfilled vs. did not fulfill any risk criterion. For EPI*bipolar*, we pooled the low-risk and the high-risk groups to provide a binary outcome. As 50% equals random classification, we considered a classification significant if the mean classification accuracy across folds minus lower confidence interval was higher than 50%. Additionally, we compared the correctly and incorrectly classified subjects to identify factors potentially influencing the achieved accuracy using *χ2* for medication (yes/no), recruitment pathway, smoking status, present/lifetime cannabis use, study site and MRI scanner used; Mann–Whitney U test for early life stress (CTQ) as CTQ was not normally distributed (Kolmogorov–Smirnov *p* < 0.001). Of note, the recruitment pathways did not automatically represent the distributions of diagnoses of ADHD and depression in the sample. Therefore, we also tested for ADHD diagnosis separately. As depressive symptoms were an inherent criterion of all three risk instruments, this comparison was not possible for depression.

## 3. Results

### 3.1. Demographics

See Table 2 and Table 3 for detailed information on socio-demographic and clinical variables among groups. The subjects who fulfilled the BD risk syndrome according to BPSS-P (Table 2) did not differ from those who did not fulfill any risk syndrome in any of the demographic variables. The subjects who fulfilled the BD risk syndrome according to BARS (Table 2) were more likely to take medication (*χ2* = 5.018, *p* = 0.025), to smoke (*χ2* = 6.529, *p* = 0.038) and to have entered the study via the depression recruitment pathway but less likely via the ADHD pathway (*χ2* = 8.823, *p* = 0.012) than those who did not fulfill any risk syndrome. The subjects categorized into the low-risk and high-risk groups according to EPI*bipolar* (Table 3) were both more likely to take medication (*χ2* = 8.077, *p* = 0.018) and to have entered the study via the depression recruitment pathway but less likely via the ADHD recruitment pathway (*χ2* = 23.707, *p* < 0.001) than those who did not fulfill any risk syndrome.

### 3.2. Statistical Analysis

There were no significant results for all LME models in the main analysis after correcting for multiple comparisons. Using BPSS-P, subjects did not show structural alterations in the total hippocampus (*F*(1, 251.458) = 0.088, *p_FDR_* = 0.872), nor in the total amygdala (*F*(1, 257.812) = 0.026, *p_FDR_* = 0.872). If categorized by EPI*bipolar* in three risk groups, there was neither a volume difference for the total hippocampus (*F*(2, 260.590) = 1.104, *p_FDR_* = 0.333), nor for the total amygdala (*F*(2, 262.751) = 3.107, *p_FDR_* = 0.092). Similarly, BARS categorization did not yield different structural volumes between risk groups for the total hippocampus (*F*(1, 253.214) = 0.541, *p_FDR_* = 0.670) nor the total amygdala (*F*(1, 255.927) = 0.182, *p_FDR_* = 0.670). For a view of descriptive volume differences between all groups, see the boxplot diagram in Appendix D.

Since there was no significant difference in the main analysis, we did not perform any post hoc tests for the total volumes and did not analyze the subfields and nuclei as part of our confirmatory hypothesis. To have a closer look at potential differences between single subfields and nuclei, we did perform LME models as an explicitly explorative analysis. Here, only the medial nucleus showed a significantly higher volume in subjects fulfilling the BPSS-P risk criterion after correcting for multiple comparisons (*F*(1, 231.662) = 13.706, *p_FDR_* < 0.05). For a detailed view of all explorative LME results, see Appendix E.

### 3.3. Machine Learning Analysis

Detailed information on the outcomes of the SVM for all three risk assessment tools can be found in Table 4. No significant predictions could be found for EPI*bipolar* and BARS. However, using the 10-fold cross-validation for BPSS-P, the SVM could significantly classify subjects at risk for BD with a balanced accuracy of 66.90% (95% *CI* 59.2–74.6), a Cohen’s kappa of 0.275 (95% *CI* 0.149–0.401), a sensitivity of 63% (95% *CI* 49.7–76.3) and a specificity of 63.0% (95% *CI* 49.7–76.3). There were no significant differences between subjects categorized at risk and those not at risk in terms of age (*df* = 262, *t* = 1.545, *p* = 0.417), sex (*df* = 1, *χ2* = 0.148, *p* = 0.7), medication (*df* = 1, *χ2* = 1.182, *p* = 0.669), recruitment pathway (*df* = 2, *χ2* = 2.215, *p* = 0.33), first-degree relatives (*df* = 1, *χ2* = 0.66, *p* = 0.797), ADHD diagnosis (*df* = 1, *χ2* = 0.673, *p* = 0.412), early life stress (Mann–Whitney *U* = 7309.5, *p* = 0.79), smoking status (*df* = 2, *χ2* = 2.575, *p* = 0.276), present cannabis use (Fisher–Freeman–Halton’s exact test *p* = 0.972), lifetime cannabis use (Fisher–Freeman–Halton’s exact test *p* = 0.730) and site (Fisher–Freeman–Halton’s exact test *p* = 0.071). The falsely and correctly classified subjects differed in scanner type (Fisher–Freeman–Halton’s exact test *p* = 0.032).

**Figure 1:**
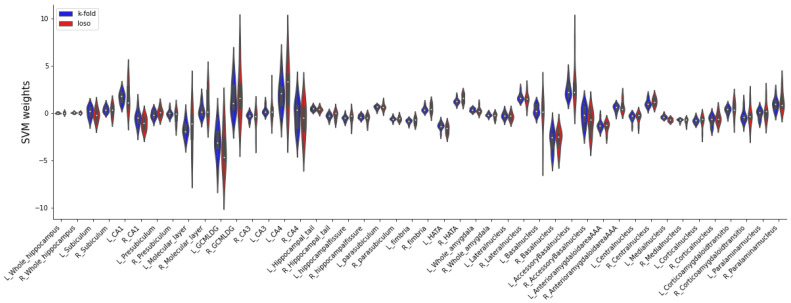
Comparison of the contribution of individual features to the SVM classification between 10-fold and leave-one-site-out classifications. The violin plots represent the SVM weights for each single feature across all folds. The similarity of both patterns suggests that the SVM relied on similar structural patterns in both cross-validation methods.

Using the leave-one-site out validation approach for BPSS-P, subjects could also be classified significantly above chance level with a balanced accuracy of 61.9% (95% *CI* 52.0–71.9), a Cohen’s kappa of 0.197 (95% *CI* 0.033–0.361), a sensitivity of 45.0% (95% *CI* 17.7–72.2) and a specificity of 78.9% (95% *CI* 60.2–97.7). There were no significant differences between subjects categorized at risk and those not at risk in terms of age (*df* = 262, *t* = 0.522, *p* = 0.602), sex (*df* = 1, *χ2* = 0.1167, *p* = 0.28), medication (*df* = 1, *χ2* = 0.051, *p* = 0.822), first degree relatives (*df* = 1, *χ2* = 1.625, *p* = 0.202), ADHD diagnosis (*df* = 1, *χ2* = 0.338, *p* = 0.561), early life stress (Mann–Whitney *U* = 7001.0, *p* = 0.375), present cannabis use (Fisher–Freeman–Halton’s exact test *p* = 0.883), lifetime cannabis use (Fisher–Freeman–Halton’s exact test *p* = 0.736), site (Fisher–Freeman–Halton’s exact test *p* = 0.08) and scanner type (Fisher–Freeman–Halton’s exact test *p* = 0.615). The falsely and correctly classified subjects (*n* = 83) differed in recruitment pathway (*df* = 2, *χ2* = 6.838, *p* = 0.033) and smoking status (*df* = 2, *χ2* = 5.953, *p* = 0.51). More specifically, the subjects recruited via the early recognition pathway were more frequently falsely classified as high-risk (35.2% versus 24.1% of all falsely classified), whereas the subjects recruited via the depression and ADHD pathways were more frequently falsely classified as no-risk (55.2% versus 46.3% and 20.7% versus 18.5%, respectively). The non-smokers were more frequently classified as high risk (48.1% versus 27.6% of all falsely classified), whereas the current smokers and past smokers were more frequently classified as no-risk (51.7% versus 40.7% and 20.7% versus 11.1%, respectively). Past or actual smoking was more frequent among the falsely classified subjects recruited via depression and ADHD pathways (23.7% early recognition, 68.1% depression, 72.2% ADHD). The distributions of model weights (i.e., contributions of individual features) among both 10-fold and leave-one-site-out approaches remained consistent (see Figure 1).

## 4. Discussion

To our best knowledge, this is the first study investigating volume differences for the hippocampus, amygdala and their subfields/nuclei in help-seeking individuals fulfilling a risk criterion for BD. We found no significant structural differences in the hippocampus and amygdala between subjects with no risk and at-risk states for BD using conventional statistics. However, we could classify the individuals fulfilling versus not fulfilling the risk criterion according to BPSS-P using machine learning (i.e., an SVM approach) with moderate performance. The exploratory analyses revealed a higher volume of the medial nucleus of the amygdala in the subjects fulfilling the BPSS-P risk criterion. The SVM classification of risk criteria according to BARS and EPI*bipolar* was not significant.

Unlike some studies in manifest BD [18,30,66], we could not identify significant volume differences in the hippocampus, amygdala and/or their subfields/nuclei in subjects fulfilling a risk criterion according to state-of-the-art risk assessment tools during confirmatory hypothesis testing. This is in accordance with studies of subjects with genetic risk for BD (i.e., first-degree relatives), which have not identified any differences in hippocampal or amygdala volumes compared to HC [6,66,67]. This might imply that individuals at risk for BD may not show structural abnormalities in those limbic regions prior to diagnosis and that the volume reduction of the hippocampus and its subfields found in manifest disorder may not be a causal mechanism for BD pathogenesis, since it seems to not happen prior to BD onset.

In contrast with group comparisons using conventional statistics, an SVM could classify the subjects fulfilling the BPSS-P risk criterion with a balanced accuracy of 66.9%, which remained significant in leave-one-site-out cross-validation. Unlike null hypothesis testing, machine learning techniques might be more appropriate for identifying discrete, multivariate differences, which are more representative of psychiatric disorders [68]. The classification performance using hippocampal subfields and nuclei of the amygdala was similar to our previous study using regional cortical thickness, cortical surface areas and volumes of subcortical structures (63.1 and 56.2% 10-fold versus leave-one-site-out) (Mikolas et al., 2023) [35]. The classification of patients with manifest BD versus HC by the ENIGMA consortium performed similarly (65.23% and 58.67% k-fold versus leave-one-site-out) [62]. At least five other studies aimed to classify first-degree relatives, healthy subjects and/or other diagnoses and achieved accuracies of 59.7 up to 83.21% [69]. However, those studies mostly used small sample sizes and, most importantly, did not perform a leave-one-site-out validation, which might favor higher accuracies [13,68,70]. Thus, our results suggest that although not detected by conventional statistical methods, structural abnormalities in limbic regions are already present in the at-risk state. These may increase after the transition to a full-blown disorder and later during the time course of BD development [17]. Although the studies in adults with BD suggest volume reductions of the amygdala and hippocampus, our exploratory analyses revealed a higher volume of the medial nucleus of the amygdala in our sample of help-seeking participants at risk. The amygdala is a dynamic region that develops continuously until early adulthood [71]. The direction and exact dynamics of structural changes still need to be understood.

Although demographic confounders seem to not have influenced the classification using the pooled sample (i.e., the 10-fold method), recruitment pathway and smoking status might have influenced the leave-one-site-out classification. Interestingly, subjects recruited via the depression and ADHD pathways were more likely to smoke or have smoked in the past, which suggests a shared effect. However, there were subjects with depression and ADHD also in the group entering the study via the early recognition pathway, which suggests that smoking status might have been the true source of this effect. Indeed, longitudinal evidence showed that smoking might lead to reduced hippocampal volumes [72]. Another confounder was the type of scanner. Seven subjects were scanned on a different scanner type (see Methods), out of which five were falsely classified. Future clinical diagnostic tools based on machine learning should include clinical data, in particular, smoking habits. Samples should include more subjects scanned using different scanner types to allow for better generalization across different scanners. Furthermore, we cannot exclude the possibility of subcortical volume differences between single risk factors, as we only tested between the overall scores of the risk assessment tools. Some risk factors, such as a family history of BD, may contribute more to a structural abnormality than other factors. Additionally, we cannot rule out that the single risk factor contributions to the achieved balanced accuracy might be non-specific to BD conversion. These factor-specific associations between BD risk and conversion should be investigated in future studies. As already stated, our achieved balanced accuracy of 66.9% for leave-one-site-out cross-validation using BPSS-P was comparable to previous ML studies classifying BD [69]. Still, this number lacks clinical utility. Since we only analyzed the baseline of the Early-BipoLife project, we could not include actual conversion rates to BD after risk assessment. These should be included as outcomes in future investigations. Compared to our previous study using regional cortical thickness, cortical surface areas and volumes of unparcelled cortical structures (Mikolas et al., 2023) [35], balanced accuracies were not much different numerically when using hippocampal subfields and amygdala nuclei as features for the SVM. The latter approach even seems to be more prone to confounders. On the other hand, the benefit of these features should be evaluated separately in future studies using longitudinal data and models should be trained to recognize subjects who transitioned to BD. Whereas the SVM classification based on BPSS-P risk assessment was above chance, the predictions using EPI*bipolar* and BARS failed, which was a similar pattern to Mikolas et al. (2023) [35]. There are several possible reasons for the better performance of BPSS-P. First, BPSS-P assigned noticeably fewer subjects to the risk group compared to both other instruments (22.3% for BPSS-P, 71.8% for BARS, 88.9% low-risk and high-risk pooled for EPI*bipolar*). The more conservative classification approach using BPSS-P seems to go along with a higher sensitivity in the detection of volume differences in limbic regions. Thus, structural abnormalities in these regions might only be traceable for a specific subgroup with a higher risk state. Additionally, subjects categorized at risk by EPI*bipolar* and BARS were more often diagnosed with unipolar depression compared to BPSS-P. This could have led to a higher heterogeneity in the high-risk sample of EPI*bipolar* and BARS, thus impeding the classification sensitivity for BD solely.

The concept of the at-risk state for BD is in development. Studies showed transition rates of 8% and 25% using different criteria with a follow-up length from 1 to 21 years compared to a cumulative lifetime incidence from 1.5% to 2% in the general population [5]. Although subjects fulfilling the risk criterion might benefit from psychiatric treatment options based on the symptoms they display, better prediction rates are necessary to aid specific clinical decisions, such as initiating a mood stabilizer. Structural MRI combined with longitudinal follow-up may improve prediction rates for conversion to manifest disease, especially through studies with a sufficient conversion ratio in subjects at risk. In this study, we had no control group in the form of HC to which the risk population could have been compared. Since this was a naturalistic study carried out at university clinics, and the recruitment pathways comprised ADHD diagnosis, depression diagnosis or people contacting early recognition centers, the sample was not representative of the general population. However, since people with several psychiatric disorders seem to show structural abnormalities, such as reduced volumes of hippocamps or amygdala, the statistical difference of these subcortical volumes might be underestimated [73,74].

## 5. Conclusions

Univariate testing did not reveal significant volume differences in hippocampal subfields and amygdala nuclei in subjects fulfilling the risk criteria for developing BD. On the other hand, machine learning differentiated between subjects fulfilling and not fulfilling the BPSS-P risk criteria with moderate performance. Parcellation of subcortical structures might identify patients who are at risk of developing BD with similar performance to cortical features. The specific benefit of using hippocampal subfields and amygdala nuclei should be evaluated in models using multimodal features and longitudinal data on conversion to BD. However, we found that an ML approach can be more sensitive in early illness detection than classic null hypothesis testing. Additionally, our results suggest that neural changes, such as subcortical abnormalities often found in manifest BD, may not merely be a consequence of the disorder but begin during the risk state.

## Figures and Tables

**Table 1:** Output volumes of the segmented subcortical structures.

Main Output	Hippocampal Subfields	Nuclei of the Amygdala
Hippocampus (total volume) Amygdala (total volume) Intracranial volume (ICV)	Hippocampal tail Subiculum Hippocampal fissure Presubiculum Parasubiculum Molecular Layer Granule cell layer of the dentate gyrus (GC ML DG) CA1 CA2/3 CA4 Fimbria Hippocampal amygdala transition area (HATA)	Lateral nucleus Basal nucleus Central nucleus Medial nucleus Cortical nucleus Accessory basal nucleus Paralaminar nucleus Corticoamygdaloid transition area Anterior amygdaloid area

Note. All parcelled volumes are given bilaterally.

**Table 2:** Socio-demographic characteristics for BPSS-P and BARS.

Risk Assessment Instrument	BPSS-P (*n* = 264)			BARS (*n* = 262)		
Risk criterion fulfilled	No	Yes	Test	No	Yes	Test
*n* (%)	205 (77.7)	59 (22.3)	*n*/a	74 (28.2)	188 (71.8)	
Female (%)	93 (45.4)	34 (57.6)	*χ2* = 2.759, *p* = 0.097	35 (47.3)	91 (48.4)	*χ2* = 0.026, *p* = 0.872
Age (SD)	24.88 (4.2)	24.54 (4.7)	*t* = −0.532, *df* = 262, *p* = 0.595	24.39 (3.7)	25.03 (4.6)	*t* = 1.075, *df* = 260, *p* = 0.283
Education high school (%)	165 (80.5)	41 (69.5)	*χ2* = 3.232, *p* = 0.072	62 (83.8)	142 (75.5)	*χ2* = 2.098, *p* = 0.148
Recruitment pathway						
Early recognition (%)	91 (44.4)	20 (33.9)	*χ2* = 2.076, *p* = 0.354	35 (47.3)	77 (41.0)	
Depression (%)	87 (42.4)	30 (50.8)		23 (31.1)	91 (48.4)	*χ2* = 8.823, *p* = 0.012 *
ADHD (%)	27 (13.2)	9 (15.3)		16 (21.6)	20 (10.6)	
Psychiatric Medication						
Yes (%)	111 (54.1)	39 (66.1)	*χ2* = 2.669, *p* = 0.102	34 (45.9)	115 (61.2)	*χ2* = 5.018, *p* = 0.025 *
Substance Use						
Smoking status Never smoked (%) Current smoker (%) Past smoker (%)	97 (47.3) 94 (45.9) 14 (6.8)	20 (33.9) 31 (52.5) 8 (13.6)	*χ2* = 4.784, *p* = 0.091	42 (56.8) 27 (36.5) 5 (6.8)	74 (39.4) 95 (50.5) 19 (10.1)	*χ2* = 6.529, *p* = 0.038 *
Cannabis present No use (%) <1×/month (%) ~1×/month (%) 2–9×/month (%) ≥10×/month (%)	147 (71.7) 17 (8.3) 12 (5.9) 15 (7.3) 14 (6.8)	45 (76.3) 3 (5.1) 2 (3.4) 2 (3.4) 7 (11.9)	*χ2* = 3.836, *p* = 0.429	61 (82.4) 4 (5.4) 3 (4.1) 3 (4.1) 3 (4.1)	129 (68.6) 16 (8.5) 11 (5.9) 14 (7.4) 18 (9.6)	*χ2* = 5.350, *p* = 0.253
Cannabis lifetime No use (%) <1×/month (%) ~1×/month (%) 2–9×/month (%) ≥10×/month (%)	84 (41.0) 46 (22.4) 9 (4.4) 24 (11.7) 42 (20.5)	23 (39.0) 11 (18.6) 2 (3.4) 6 (10.2) 17 (28.8)	*χ2* = 1.977, *p* = 0.740	38 (51.4) 16 (21.6) 2 (2.7) 7 (9.5) 11 (14.9)	68 (36.2) 40 (21.3) 9 (4.8) 24 (12.8) 47 (25.0)	*χ2* = 6.532, *p* = 0.163

* *p*  ≤  0.05.

**Table 3:** Socio-demographic characteristics for *EPIbipolar*.

Risk Assessment Instrument	EPIb*ipolar* (*n* = 271)			
Risk criterion fulfilled	No-risk	Low-risk	High-risk	Test
*n* (%)	30 (11.1)	136 (50.2)	105 (38.7)	*n*/a
Female (%)	10 (33.3)	62 (45.6)	57 (54.3)	*χ2* = 4.550, *p* = 0.103
Age (SD)	24.13 (3.03)	25.40 (4.61)	25.02 (4.34)	*F* = 0.570, *df* = 2, *p* = 0.566
Education high school (%)	24 (80.0)	107 (78.7)	81 (77.1)	*χ2* = 0.144, *p* = 0.931
Recruitment pathway				
Early recognition (%)	14 (46.7)	50 (36.8)	51 (48.6)	*χ2* = 23.707, *p* < 0.001 **
Depression (%)	5 (16.7)	72 (52.9)	43 (41.0)	
ADHD (%)	11 (36.7)	14 (10.3)	11 (10.5)	
Psychiatric Medication				
Yes (%)	11 (36.7)	87 (64.0)	57 (54.3)	*χ2* = 8.077, *p* = 0.018 *
Substance Use				
Smoking status Never smoked (%) Current smoker (%) Past smoker (%)	16 (53.3) 9 (30.0) 5 (16.7)	64 (47.1) 64 (47.1) 8 (5.9)	38 (36.2) 56 (53.3) 11 (10.5)	*χ2* = 8.771, *p* = 0.067
Cannabis present No use (%) <1×/month (%) ~1×/month (%) 2–9×/month (%) ≥10×/month (%)	25 (83.3) 1 (3.3) 0 (0.0) 2 (6.7) 2 (6.7)	96 (70.6) 11 (8.1) 8 (5.9) 8 (5.9) 13 (9.6)	76 (72.4) 10 (9.5) 6 (5.7) 7 (6.7) 6 (5.7)	*χ2* = 4.647, *p* = 0.795
Cannabis lifetime No use (%) <1×/month (%) ~1×/month (%) 2–9×/month (%) ≥10×/month (%)	14 (46.7) 8 (26.7) 0 (0.0) 3 (10.0) 5 (16.7)	52 (38.2) 29 (21.3) 6 (4.4) 17 (12.5) 32 (23.5)	44 (41.9) 21 (20.0) 5 (4.8) 11 (10.5) 24 (22.9)	*χ2* = 3.173, *p* = 0.923

Note. * *p* ≤ 0.05; ** *p* ≤ 0.01.

**Table 4:** Performance metrics of the linear SVM classification for all three risk assessment tools.

	Cohen’s Kappa (%)			Balanced Accuracy (%)			Sensitivity (%)			Specificity (%)		
		95% CI			95% CI			95% CI			95% CI	
		Lower	Upper		Lower	Upper		Lower	Upper		Lower	Upper
BPSS-P												
10-fold	0.275	0.149	0.401	66.9	59.2	74.6	63.0	49.7	76.3	63.0	49.7	76.3
Leave-one-site-out	0.197	0.033	0.361	61.9	52.0	71.9	45.0	17.7	72.2	78.9	60.2	97.7
BARS												
10-fold	−0.001	−0.132	0.129	49.2	41.9	56.5	56.2	44.2	68.3	42.1	35.7	48.5
Leave-one-site-out	−0.000	−0.103	0.103	48.2	40.1	56.2	53.8	41.9	65.7	42.5	30.7	54.4
EPI*bipolar*												
10-fold	−0.049	−0.143	0.045	45.0	35.3	54.8	66.8	58.0	75.5	23.3	7.2	39.4
Leave-one-site-out	−0.027	−0.203	0.148	46.2	35.6	56.8	62.4	44.4	80.5	30.0	15.2	44.8

**Figure A1:**
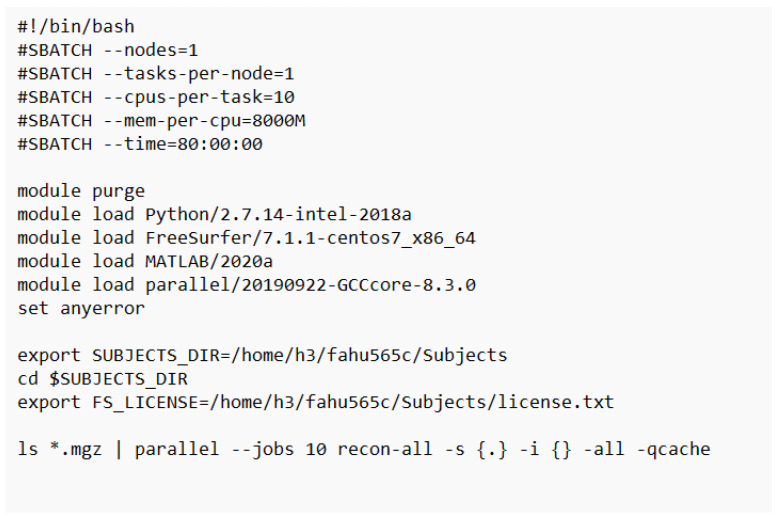
Bash shell script in a virtual Linux environment at the high-performance computing (ZIH), prompting a parallel preprocessing of the subcortical segmentation procedure.

**Figure A2:**
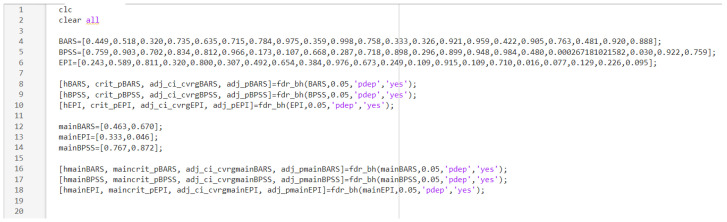
MATLAB script for calculating the FDR of the explorative analysis.

**Figure A3:**
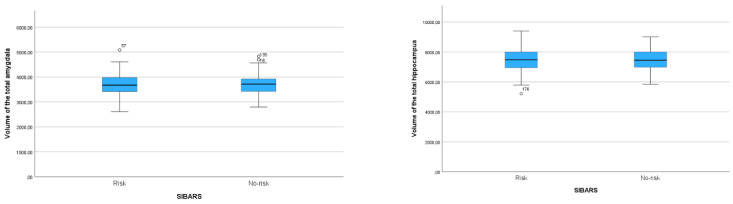

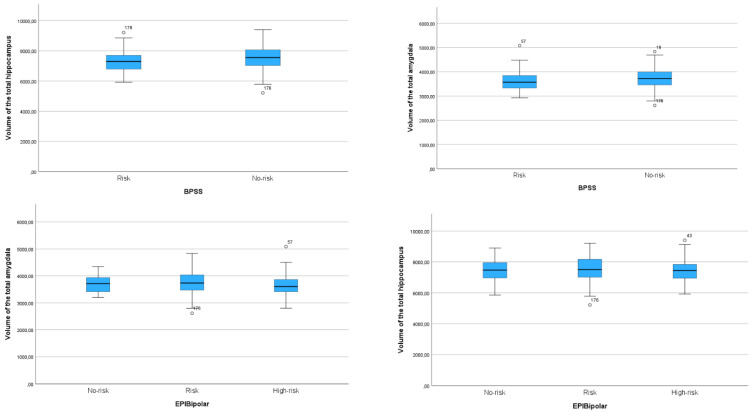
Boxplot diagrams comparing the volumes of the total hippocampus and of the total amygdala for all three risk assessment tools.

## Data Availability

Data are available upon reasonable request.

## Appendix A. Inclusion and Exclusion Criteria for the Three Recruitment Pathways

Inclusion criteria for each pathway:

1. Youth and young adults consulting early recognition centres/facilities:
  - Age: 15 to 35 years
  - Consultation of an early recognition centre/facility
  - Presence of at least one of the proposed risk factors for bipolar disorder: Family history of bipolar disorder, (sub)threshold affective symptomatology/depressive syndrome, hypomanic/mood swings, disturbances of circadian rhythm/sleep, other clinical hints
2. Young individuals with diagnosed depression:
  - Age: 15 to 35 years
  - In- or outpatients with a depressive syndrome in the context of major depressive disorder, dysthymic disorder, cyclothymic disorder, minor depressive disorder, recurrent brief depressive disorder, adjustment disorder with depressed mood, depressive disorder Not Otherwise Specified (NOS)
3. Patients with ADHD:
  - Age: 15 to 35 years
  - In- or outpatients with a clinically confirmed ADHD diagnosis

Exclusion criteria for all three pathways:

- Diagnosis of bipolar disorder, schizoaffective disorder, schizophrenia
- Diagnosis of anxiety, obsessive–compulsive or substance dependence disorder that fully explains the whole symptomatology
- Limited ability to comprehend the study
- Implied expressed negative declaration of intent to participate in the study by a minor and
- Acute suicidality

## Appendix E

**Table A1:** LME results for the explorative analysis of all twelve hippocampal subfields and nine nuclei of the amygdala for all three risk assessment tools.

	BPSS-P	BARS	EPI*bipolar*
Hippocampal subfields			
CA1	*F*(1, 258.392) = 0.015, *p* = 0. 984	*F*(1, 253.719) = 0.418, *p* = 0.998	*F*(2, 261.014) = 1.423, *p* = 0.581
CA2/3	*F*(1, 257.737) = 0.057, *p* = 0. 984	*F*(1, 253.306) = 0.226, *p* = 0.998	*F*(2, 260.560) = 0.531, *p* = 0.877
CA4	*F*(1, 256.403) = 0.002, *p* = 0. 984	*F*(1, 252.530) = 0.133, *p* = 0.998	*F*(2, 260.047) = 0.210, *p* = 0.896
Molecular layer	*F*(1, 257.835) = 0.016, *p* = 0. 984	*F*(1, 253.161) = 0.942, *p* = 0.998	*F*(2, 260.538) = 1.143, *p* = 0.611
GC ML DG	*F*(1, 256.400) = 0.044, *p* = 0. 984	*F*(1, 252.529) = 0.115, *p* = 0.998	*F*(2, 260.057) = 0.224, *p* = 0.896
Hippocampal tail	*F*(1, 257.433) = 1.866, *p* = 0. 908	*F*(1, 255.414) = 0.075, *p* = 0.998	*F*(2, 262.303) = 1.186, *p* = 0.611
Subiculum	*F*(1, 258.919) = 0.094, *p* = 0. 984	*F*(1, 253.869) = 0.574, *p* = 0.998	*F*(2, 261.239) = 0.712, *p* = 0.795
Presubiculum	*F*(1, 258.126) = 0.146, *p* = 0. 984	*F*(1, 252.960) = 0.994, *p* = 0.998	*F*(2, 260.245) = 0.425, *p* = 0.877
Parasubiculum	*F*(1, 252.592) = 0.131, *p* = 0. 984	*F*(1, 254.835) = 0.095, *p* = 0.998	*F*(2, 260.532) = 0.962, *p* = 0.672
Fimbria	*F*(1, 238.399) = 0.184, *p* = 0. 984	*F*(1, 256.843) = 0.844, *p* = 0.998	*F*(2, 262.917) = 0.024, *p* = 0.976
Hippocampal fissure	*F*(1, 258.786) = 2.610, *p* = 0. 749	*F*(1, 254.065) = 0.001, *p* = 0.998	*F*(2, 261.224) = 0.397, *p* = 0.877
HATA	*F*(1, 259) = 1.138, *p* = 0. 984	*F*(1, 254.478) = 0.000, *p* = 0.998	*F*(2, 261.619) = 1.397, *p* = 0.581
Nuclei of the amygdala			
Cortical ncl.	*F*(1, 247.747) = 4.766, *p* = 0. 315	*F*(1, 256.286) = 0.498, *p* = 0.998	*F*(2, 262.740) = 2.239, *p* = 0.452
Medial ncl.	*F*(1, 231.662) = 13.706, *p* = 0.0056 **	*F*(1, 256.062) = 0.091, *p* = 0.998	*F*(2, 262.792) = 0.089, *p* = 0.961
Basal ncl.	*F*(1, 257.337) = 0.016, *p* = 0. 984	*F*(1, 255.524) = 0.010, *p* = 0.998	*F*(2, 262.493) = 2.233, *p* = 0.452
Central ncl.	*F*(1, 259) = 0.500, *p* = 0. 984	*F*(1, 257) = 0.014, *p* = 0.998	*F*(2, 265) = 0.342, *p* = 0.877
Lateral ncl.	*F*(1, 252.575) = 1.096, *p* = 0. 984	*F*(1, 255.551) = 0.970, *p* = 0.998	*F*(2, 262.244) = 4.214, *p* = 0.336
Accessory basal ncl.	*F*(1, 249.097) = 0.004, *p* = 0. 984	*F*(1, 256.320) = 0.003, *p* = 0.998	*F*(2, 263.123) = 2.586, *p* = 0.452
AAA	*F*(1, 233.022) = 0.000, *p* = 0. 984	*F*(1, 256.675) = 0.648, *p* = 0.998	*F*(2, 263.346) = 2.066, *p* = 0.452
Corticoamygdaloid transition area	*F*(1, 247.829) = 0.009, *p* = 0. 984	*F*(1, 255.937) = 0.010, *p* = 0.998	*F*(2, 262.786) = 1.497, *p* = 0.581
Paralaminar ncl.	*F*(1, 256.680) = 0.095, *p* = 0. 984	*F*(1, 255.558) = 0.020, *p* = 0.998	*F*(2, 262.455) = 2.375, *p* = 0.452

** *p* ≤ 0.01. All *p*-values were corrected for multiple comparisons using FDR.
